# Assessment of the Effects of Inspiratory Muscle Training (IMT) and Aerobic Training on the Quality of Life of Patients with Chronic Obstructive Pulmonary Disease

**Published:** 2019-03

**Authors:** Amir Hossein Abedi Yekta, Mehrshad Poursaeid Esfahani, Shahin Salehi, Mohammad Hassabi, Shahrzad Khosravi, Shahram Kharabian, Mohammad Reza Sohrabi, Amir Ali Mafi, Saeed Rezaei

**Affiliations:** 1 Department of Sports Medicine, School of Medicine, Shahid Beheshti University of Medical Sciences, Tehran, Iran,; 2 Clinical Research Development Center of Loghman Hakim Hospital, Shahid Beheshti University of Medical Sciences, Tehran, Iran.

**Keywords:** Chronic obstructive pulmonary disease, Inspiratory muscle training, Aerobic exercise, Quality of life

## Abstract

**Background:**

Chronic obstructive pulmonary disease (COPD) is a major cause of morbidity and mortality worldwide. The aim of this study was to investigate the effects of inspiratory muscle training (IMT) and aerobic exercise on health-related quality of life of patients with COPD.

**Materials and Methods:**

This randomized controlled clinical trial was conducted on 60 patients with moderate to severe COPD, who were referred to Imam Hossein Hospital of Tehran, Iran in 2016. The patients were randomly assigned to four groups and treated for eight weeks. Group 1 (n=16) participated in 16 sessions of IMT (15 minutes per session), group 2 (n=14) performed aerobic exercises twice a week (40 minutes per session), group 3 (n=15) performed IMT and aerobic exercises, and group 4 (n=15) received no intervention, except for routine treatments (control). Quality of life was evaluated based on the Saint George’s Respiratory Questionnaire (SGRQ) at baseline, week 4, and week 8 after the intervention.

**Results:**

After eight weeks, all four groups experienced a significant improvement in their quality of life (P<0.05), and group 3 (IMT and aerobic exercise) showed the greatest improvement. However, quality of life improvement in group 4 (control) was less than the other three groups (P<0.05).

**Conclusion:**

Aerobic exercise and IMT were more effective than routine protocols in improving the quality of life of COPD patients. Furthermore, short-term IMT plus aerobic exercise had the greatest impact on improving the health-related quality of life of COPD patients and could be used in the management of these patients.

## INTRODUCTION

Chronic obstructive pulmonary disease (COPD) is a set of physiological disorders associated with the obstruction of airways (1). The characteristic feature of COPD is persistent airway obstruction that is usually progressive (2). The most common sign of COPD is dyspnea, which limits exercise and physical activity in patients and leads to chronic avoidance behaviors towards physical activity (3). COPD affects 4–6% of the world’s population and is one of the leading causes of mortality worldwide. It is also the fourth cause of mortality in the United States (4).

Due to peripheral airway obstruction in COPD, air volume may be trapped in the lungs (hyperinflation). The respiratory rate may be increased because inspiration occurs before the air is released from the lungs. Moreover, rapid shallow breathing leads to respiratory muscle fatigue and inefficient gas exchange; therefore, patients with COPD show signs of dyspnea (5). Previous studies have shown that dyspnea is associated with symptoms of depression, anxiety, fatigue, sleep disturbances, pain, and decreased quality of life (QOL) (6, 7).

Patients with COPD show an initial increase in lactic acidosis during exercise and are unable to meet their ventilation needs. Therefore, interventions that improve ventilation, such as inspiratory muscle training (IMT), have the potential to reduce dyspnea and improve exercise tolerance in these patients. On the other hand, low levels of physical activity play a role in deconditioning of skeletal muscles and reducing exercise tolerance, which negatively affects QOL (4). Considering the insidious and progressive nature of COPD, pulmonary function may be easily lost by 50% before the emergence of symptoms. Sudden acute exacerbation of symptoms can affect the patients’ QOL and treatment costs. Therefore, the main goal of COPD management and treatment is to improve the symptoms and QOL (5).

Pulmonary rehabilitation, including sports training, education, nutritional interventions, and mental support, is the standard care for patients with COPD (8). Respiratory exercises using IMT and exercise programs are components of pulmonary rehabilitation and are used to improve respiratory function and COPD management. In recent years, there have been few studies on the use of IMT in COPD patients. A meta-analysis of randomized controlled trials (RCTs) in patients with COPD revealed that IMT, as an independent treatment, improves the function (strength and endurance) of respiratory muscles, alleviates the symptoms of dyspnea, and improves exercise tolerance (9).

Aerobic exercise training is considered an essential component of pulmonary rehabilitation (10). Also, measurement of QOL is important for clinical decision-making. It has been observed that respiratory exercises improve QOL and dyspnea (11, 12). Nevertheless, the effectiveness of IMT as a complement to general exercise remains uncertain. Although IMT results in significant improvements in respiratory muscle performance, its effects on pulmonary function, clinical outcomes, and QOL have not been confirmed by scientific evidence (13, 14).

Despite the cost-effective and non-invasive nature of exercise therapy and IMT, there are limited studies on the effectiveness of these methods in improving the QOL of patients with COPD. Therefore, the aim of this study was to determine the effectiveness of conventional aerobic exercises and respiratory muscle training in improving the QOL of patients with COPD.

## MATERIALS AND METHODS

This RCT was performed on 60 patients with moderate to severe COPD, who were referred to Imam Hossein Hospital of Tehran, Iran in 2017. The patients entered the study after the researchers explained the timing, protocol, objectives, and methods of the study. Written informed consents were also obtained from the participants. This clinical trial was registered in the Iranian Registry of Clinical Trials (registration number: IRCT20180205038633N1).

Spirometry was performed for all patients, using COSMOS MetaSoft 2000, Germany. The patients were enrolled if they had stage 2 or 3 COPD according to the Global Initiative for Chronic Obstructive Lung Disease (GOLD) criteria (Table 1), which categorize airflow limitations into different stages. Other inclusion criteria were as follows: age range of 30–70 years; no previous known diseases, such as heart disease (e.g., congestive heart failure and coronary artery disease), renal disease (e.g., end stage renal disease and chronic renal failure), or liver disease (e.g., hepatic cirrhosis and hepatic cancer); no history of known pulmonary diseases, such as lung cancer and pleural disease; no history of musculoskeletal diseases (e.g., myasthenia gravis) or restrictive deformities of the lungs; lack of severe limitations in the limbs inhibiting aerobic exercise; no pulmonary surgery in the past 12 months; no recent fracture of the ribs in the past six months; no history of psychotropic diseases; and no use of drugs, alcohol, or psychiatric drugs.

**Table 1.** Demographic data and patients’ characteristics^*^

**Variable**	IMT+ Aerobic Exercise (n=15)	Aerobic Exercise (n=14)	IMT (n=16)	Control (n=15)	P-value^**^
**Age (year)**	51.33±10.4	53.5±10.37	51.88±9.05	55.67±11.08	0.652
**Sex**	Female: 6 (40)	Female: 6 (42.9)	Female: 9 (56.3)	Female: 8 (53.3)	0.768
	Male: 9 (60)	Male: 8 (57.1)	Male: 7 (43.8)	Male: 7 (46.7)	
**Weight (kg)**	78.47±13.6	74.57±12.8	74.94±12.3	70.07±16.4	0.437
**Height (cm)**	168.27±7.9	165.5±11.68	166.7±8.94	163.5±8.07	0.542
**BMI (kg/m^2^)**	27.6±3.7	27.39±5.11	27.05±4.53	25.98±4.1	0.752
**Type of disease**	CB: 11 (73.3)	CB: 10 (71.4)	CB: 13 (81.3)	CB: 12 (80)	0.899
	E: 4 (26.7)	E: 4 (28.6)	E: 3 (18.7)	E: 3 (20)	
**Disease severity**	Moderate: 6 (60)	Moderate: 7 (50)	Moderate: 8 (50)	Moderate: 7 (46.7)	0.896
	Severe: 9 (40)	Sever: 7 (50)	Sever: 8 (50)	Sever: 8 (53.3)	
**Duration of disease**	8.53±3.38	12.21±7.19	10.19±4.55	14.2±7.33	0.055

IMT: Inspiratory muscle training; BMI: Body mass index; CB: Chronic bronchitis; E: Emphysema.

* Data are presented as mean±SD or frequency (%)

** Significance at p≤0.05.

On the other hand, the exclusion criteria were as follows: exacerbation of the disease during the study; need for long-term oxygen therapy for more than 15 hours a day; and occurrence of complications, such as pneumothorax or diseases exacerbating and disrupting treatment. All patients were under complete clinical observation (e.g., weight, height, and BMI). Information about the type of disease, severity of the disease, and duration of the disease was also recorded.

### Intervention

Patients were randomly divided into four groups with an age adjustment of 10 years (from 30 to 69 years) and were treated for eight weeks. A total of 68 patients were divided into four groups, each consisting of 17 patients. After the follow-up and post-training assessment, eight patients were removed from the study due to loss to follow-up. Finally, 60 patients were included in the study. The first group (n=16) received IMT treatment, the second group (n=14) performed aerobic exercises, and the third group (n=15) received combination therapy, including breathing exercises, followed by aerobic exercises of lower extremities. The fourth group (n=15) received no specific interventions, except for the usual treatments (control group).

Aerobic exercise was performed on a treadmill and a foot ergometer two days a week with 40–60% of heart rate reserve (HRR) for 40 minutes per session. The first five minutes of each session included low-speed warm-up, and the last five minutes included low-speed cool-down. In the group with respiratory training, the intensity of training was 40–60% (S-Index: 40–60%). Training was performed two days a week, and in each session, repetitive IMT (5 sets of 15–30 repetitions) was performed for about 15 minutes(S-Index: 40–60%).Additionally, all patients were advised to perform aerobic exercises, including mild to moderate walking at home.

### Patient evaluation

The standard Saint George’s Pulmonary Disease Questionnaire (SGRQ) (Table 2) was used to assess the QOL of patients with airway obstruction. All patients were evaluated at the beginning of treatment, in the fourth week, and at the end of treatment (eighth week). The questionnaire was completed by the patients under the researcher’s supervision. The total SGRQ score was measured for each patient. The questionnaire consisted of 50 questions, including the symptoms, activity, and impact on daily life. The patient’s scores ranged from 0 to 100, with higher scores indicating lower QOL. A change of four units in the total score of the questionnaire was considered significant. In this study, due to cultural reasons, the Persian version of SGRQ was used and validated. The reliability, validity, and specificity of this questionnaire for evaluating patients with COPD have been confirmed in Iran (15, 16).

**Table 2.** Changes in the quality of life index according to the SGRQ questionnaire in COPD patients

**Group**	Baseline (mean±SD)	4 week (mean±SD)	8 week (mean±SD)	Difference	P-value^**^
**Control (n=15)**	21.87 ± 4.49	19.96 ± 5.08	17.68 ± 4.34	4.19±3.28	0.0001
**IMT (n=16)**	17.9 ± 4.98	15.84 ± 3.72	13.1 ± 5.2	4.79±5.55	0.002
**Aerobic Exercise (n=14)**	19.01 ± 4.81	16.25 ± 4.5	13.93 ± 4.7	5.07±5.48	0.0001
**IMT+ Aerobic Exercise (n=15)**	15.53 ± 5.64	12.12 ± 5.51	10 ± 6.07	5.5±3.54	0.0001

COPD= Chronic obstructive pulmonary disease; SGRQ= St.George Respiratory Questionnaires; SD = standard deviation.

**Figure 1.**
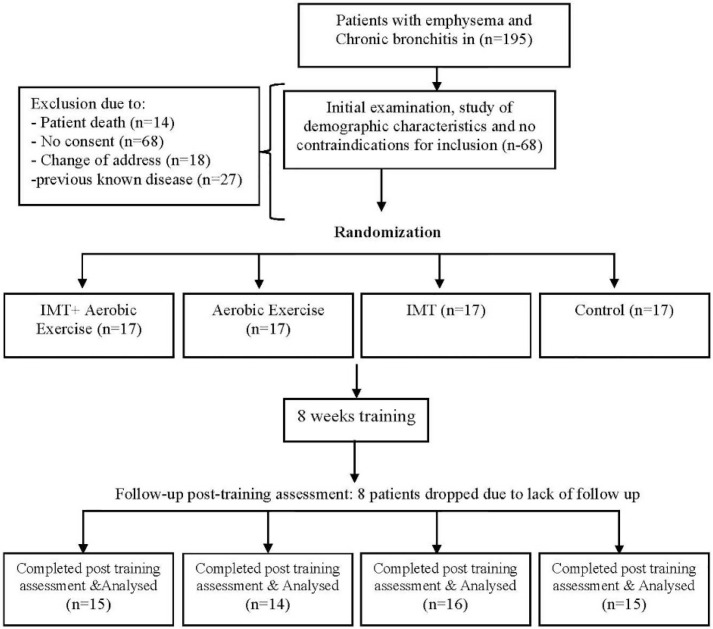
The study flowchart

### Statistical analysis

For statistical analysis, SPSS version 22 was used. Data were analyzed by descriptive statistics, including frequency, mean, standard deviation, and percentage of frequency, as well as inferential statistics, including Chi-square, ANOVA, and non-parametric Friedman and Wilcoxon tests. The significance level was considered to be 0.05.

## RESULTS

In this study, 29 women and 31 men, with the mean age of 53.07±10.11 years (range: 32–70 years), were recruited. The demographic and clinical characteristics of the patients are presented in Table 1. There were no significant differences in terms of age, sex, height, weight, BMI, type of disease, duration of disease, or severity of disease between the four treatment groups (P>0.05).

The results of SGRQ in COPD patients showed that QOL improved in all four groups at the end of the fourth and eighth weeks (P<0.05). The findings showed that aerobic exercise and IMT led to the greatest improvement in QOL (5.5±3.54); the least improvement was reported in the control group (3.28±4.19) (Table 2).

## DISCUSSION

Currently, scientific evidence regarding the impact of various treatments, including IMT, on COPD patients is somewhat contradictory. In view of the increasing prevalence of COPD and its high mortality and morbidity, the present study aimed at identifying the best treatment options by evaluating the effects of IMT and aerobic exercise on the QOL of patients with COPD. There were no significant differences between the four treatment groups regarding age, sex, weight, height, BMI, type of disease (chronic bronchitis/emphysema), severity of disease, or duration of disease, which indicates the ineffectiveness of these variables on the results.

The results of SGRQ showed a significant improvement in the QOL of all three treatment groups. The overall score of QOL in the control group also showed a significant improvement. In this study, the greatest effects on QOL were reported in the IMT plus aerobic exercise group, aerobic exercise group, IMT group, and control group, respectively. Since all patients, including the control group, were advised to perform mild to moderate aerobic exercises during the week for ethical reasons, walking and possibly sports exercises can explain the significant improvement of QOL in the control group. Improvements observed in the control group also showed that routine therapies can be somewhat effective.

In a study by Ahmed et al. from Egypt, the efficacy of aerobic exercise plus IMT on the QOL of COPD patients was higher than aerobic exercise alone for two months. However, both methods had significant effects on the improvement of QOL (4), which is consistent with the findings of the present study. Moreover, Chuang et al. in a study from Taiwan showed that IMT (five sessions per week for eight weeks) had significant effects on improving the QOL of patients with moderate to severe COPD (17). Moreover, Tout et al. reported a significant improvement in all parameters of QOL (based on the score of SGRQ) in COPD patients participating in IMT, expiratory muscle training (EMT), and aerobic exercises of lower extremities for eight weeks (18); the findings of the present study confirm these results. Therefore, IMT exercises can reduce the problems of daily activities in patients with moderate to severe COPD.

The effectiveness of IMT program in the QOL of COPD patients has been also reported in other studies. A meta-analysis by Gosselink et al. showed that IMT improves the QOL (+3.8 units) of patients with COPD (19). Moreover, Geddes et al. showed a significant improvement in the QOL of adult patients with COPD after IMT (20). Beckerman et al. also showed that the IMT program significantly improved the QOL of patients with COPD (21). Additionally, Borge et al. showed the high efficacy of respiratory muscle training in improving the QOL of COPD patients (22). Another study by Charususin et al. found that IMT for three months improved QOL associated with health and physical activity in COPD patients (12).

Furthermore, Bashirian et al. showed that eight weeks of lower limb aerobic exercise had greater efficacy in improving the QOL of patients with chronic bronchitis, compared to respiratory exercises (23); these results are consistent with the findings of the present study. Moreover, Bingisser et al.(24) and Hernandez et al.(25) showed that low-intensity aerobic exercise improves the QOL of patients with COPD. These results are consistent with the findings of the present study. Moreover, the results of a study by Beaumont et al. (26) showed that IMT with threshold devices improved the inspiratory muscle strength, exercise capacity, and QOL, while reducing dyspnea; these results are in line with the present findings.

The current study is one of the few studies comparing the effectiveness of aerobic exercise with IMT in the treatment of moderate to severe COPD. This study provided valuable information regarding the effectiveness of these two methods in improving the QOL of COPD patients, which is consistent with the results of many previous studies. Therefore, treatment plans used in this study can be applied for COPD patients.

On the other hand, the present study had some limitations. First, only the short-term effects of aerobic exercise and IMT were investigated, while mid-term and long-term effects of these treatments were not investigated. Second, we included a small number of samples in each group, as it was not possible to examine more samples because of time limitations. Third, a large number of samples were eliminated from the study (unwilling to cooperate for a variety of reasons), which was beyond the researchers’ control. Finally, the limited number of treatment sessions is another limitation of this study; in fact, by increasing the number of therapeutic sessions, better treatment outcomes can be achieved.

## CONCLUSION

The results of the present study showed that all three treatments (for eight weeks) could improve the QOL of patients with COPD. However, the effectiveness of aerobic exercise plus IMT was greater than the control group. Also, combination of aerobic exercise with IMT had the greatest effects on the QOL of patients. Considering the high efficacy of aerobic exercise and IMT in improving the QOL of patients with moderate to severe COPD and the low-cost, non-invasive, and non-pharmaceutical nature of these methods, we can take an effective step in improving the health and QOL of these patients by encouraging them to use these methods. However, since limited studies have compared aerobic exercise, IMT, and combination of these two methods in the treatment of COPD patients, it is necessary to conduct more studies in the future to identify the best treatment option.
